# Human Microbiota of the Argentine Population- A Pilot Study

**DOI:** 10.3389/fmicb.2016.00051

**Published:** 2016-02-01

**Authors:** Belén Carbonetto, Mónica C. Fabbro, Mariela Sciara, Analía Seravalle, Guadalupe Méjico, Santiago Revale, María S. Romero, Bianca Brun, Marcelo Fay, Fabián Fay, Martin P. Vazquez

**Affiliations:** ^1^Genomics and Bioinformatics, Instituto de Agrobiotecnología de Rosario – CONICETRosario, Argentina; ^2^Centro de Diagnostico Medico de Alta Complejidad (CIBIC)Rosario, Argentina

**Keywords:** human microbiome, dysbiosis, South America, healthy microbiota, amplicon sequencing

## Abstract

The human microbiota is the collection of microorganisms living in or on the human body. An imbalance or dysbiosis in these microbial communities can be associated with a wide variety of human diseases (Petersen and Round, 2014; Pham and Lawley, 2014; Zaura et al., 2014). Moreover, when the microbiota of the same body sites is compared between different healthy individuals, specific microbial community features are apparent (Li et al., 2012; Yatsunenko et al., 2012; Oh et al., 2014; Relman, 2015). In addition, specific selective pressures are found at distinct body sites leading to different patterns in microbial community structure and composition (Costello et al., 2009; Consortium, 2012b; Zhou et al., 2013). Because of these natural variations, a comprehensive characterization of the healthy microbiota is critical for predicting alterations related to diseases. This characterization should be based on a broad healthy population over time, geography, and culture (Yatsunenko et al., 2012; Shetty et al., 2013; Leung et al., 2015; Ross et al., 2015). The study of healthy individuals representing different ages, cultural traditions, and ethnic origins will enable to understand how the associated microbiota varies between populations and respond to different lifestyles. It is important to address these natural variations in order to later detect variations related to disease.

## Methods

### Sampling

Samples were swabbed in a non-invasive manner from palatine tonsils, saliva, buccal mucosa, throat, and anterior nares; gastrointestinal tract samples were collected from stool samples. Twenty healthy subjects, men and women between 20 and 50 years-old living in Rosario city in the central region of Argentina, were recruited. Subjects donated blood to examine the presence of viral markers and metadata was collected by medical examination in order to select healthy individuals (**Supplementary Table S1**, also see Supplementary material for inclusion/exclusion criteria of healthy subjects). Samples were collected between May and August 2013 in a single visit and sent immediately to the lab where they were processed.

### Ethics, Consent, and Permissions

The study was approved by the Institutional Ethics Committee from the Hospital Italiano Garibaldi in Rosario, Argentina. Moreover, a consent form with information about the study, including the rights, risks, and benefits involved in participating in the study was signed by each individual.

### DNA Extraction and Sequencing

Total genomic DNA was extracted from 200 mg of each fresh stool sample using QIAamp (Qiagen, Valencia, CA, USA) DNA Stool Mini Kit following manufacturer’s instructions. Palatine tonsils, saliva, buccal mucosa, throat, and anterior nares swabs were resuspended in 200 μl sterile saline solution. Genome DNA was extracted from this solution using QIAamp (Qiagen, Valencia, CA, USA) DNA Mini Kit following manufacturer’s instructions.

For the construction of pyrotag libraries the V1–V3 hyper variable regions of the 16S rRNA gene was amplified using the 27F (5′-AGAGTTTGATCCTGGCTCAG-3′) and 534R (5′-ATTACCGCGGCTGCTGG-3′) tagged primers (Consortium, 2012a). Samples were amplified using two rounds of PCR: a first round to amplify the 16S rRNA gene (30 cycles) and a second round to add barcodes for sample identification (10 cycles). PCR reactions were performed following the procedures detailed in Rascovan et al. (2013). Duplicated reactions were performed in both rounds of PCR to reduce amplification biases and then pooled. Amplicons were cleaned using Ampure DNA capture beads (Agencourt- Beckman Coulter, Inc.), quantified using Quant-iT PicoGreen dsDNA Kit (Invitrogen Molecular Probes, Inc., Eugene, OR, USA) and pooled in equimolar concentrations before sequencing on a Genome Sequencer FLX (454-Roche Applied Sciences) using 454 GS FLX+ chemistry according to the manufacturer’s instructions.

### Amplicon Sequence Processing, OTU Classification, and Taxonomic Assignment

We chose the HMP dataset to compare with our data since it is the most complete reference collection of 16S ribosomal RNA gene sequences collected from sites across the human body (Consortium, 2012a). We are aware that the DNA extraction method used by the HMP is different from the used in this project (MoBio PowerSoil DNA Isolation Kit vs. QIAamp DNA kits). Nevertheless, recent results showed that both DNA extraction methods are reproducible enough for the analysis of bacterial community diversity of human samples (Wagner Mackenzie et al., 2015). Taking this into account we decided to use the HMP data for our comparison demonstration. The HMP dataset based on the amplification of the V1–V3 hyper variable regions of the 16S rRNA gene was downloaded from the HMP website^2^. Our dataset and the HMP dataset were processed using the QIIME v1.8 analysis pipeline (Caporaso et al., 2010b). For comparative purposes the same number of individuals (*N* = 20) was selected randomly from the HMP dataset. A random selection of 1000 reads per sample was done using a custom-made script. The command split libraries.py was used for demultiplexing and quality filtering. Reads with more than 10 mismatches in the forward primer sequence and more than eight mismatches in the reverse primer sequence were removed. Up to two mismatches were allowed in barcode sequences. Homopolymers longer than 6 bp were excluded. The mean qual score used was 25Q. The size of quality score window was set up to 50 bp. If the average score of a continuous set of 50 nucleotides fell below 25Q, the sequence was discarded. Minimum sequence length allowed was 200 bp. Filtered sequences were then clustered into operational taxonomic units (OTUs) using the pick_otus.py script with the Uclust method at 97% sequence similarity (Edgar, 2010). OTU representative sequences were aligned using PyNast algorithm with QIIME default parameters (Caporaso et al., 2010a). Phylogenetic trees containing the aligned sequences were then produced using FastTree (Price et al., 2009). Richness alpha diversity metrics and rarefaction curves were calculated by sub-sampling the OTU tables at different depths and counting the resulting number of phylotypes using 10 iterations per sample. Phylogeny-based beta diversity distances between OTUs were calculated using weighted Unifrac (Lozupone and Knight, 2005; Lozupone et al., 2007). Taxonomic classification of sequences was done with Ribosomal Database Project (RDP) Classifier using the Greengenes V13.5 database and a 50% confidence threshold (Wang et al., 2007).

### Numerical Analyses

Unifrac phylogenetic pairwise distances among samples were visualized with principal coordinates analysis (PCoA). Analysis of similarity statistics (ANOSIM) was calculated to test *a priori* sampling groups. Mann–Whitney non-parametric tests were performed to elucidate differences in taxa abundances. All calculations were carried out with R packages ‘BiodiversityR’ and ‘Vegan.’

## Results

### Argentine Human Microbiota Overview

**FIGURE 1:**
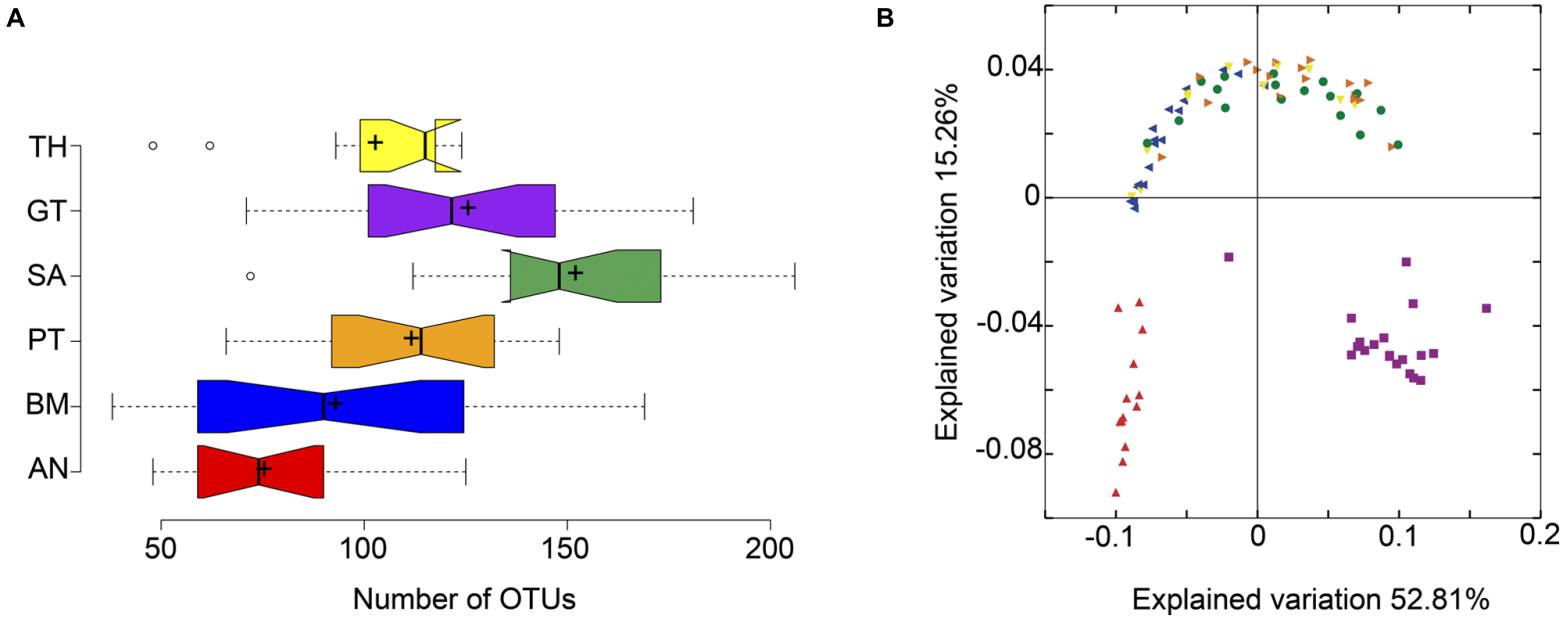
**Diversity analysis of the Argentine human microbiota.** **(A)** Alpha diversity analysis based on OTU richness, BM, buccal mucosa; TH, throat; PT, palatine tonsils; AN, anterior nares; SA, saliva; GT, gut. **(B)** Beta diversity analysis based on weighted Unifrac pairwise distances, each color represents the same body part as in **(A)**.

Results showed that OTU richness differed between body habitats. Saliva presented the higher richness and the anterior nares was the less diverse site (**Figure 1A**). Beta diversity also revealed differences in community structure between body habitats (**Figure 1B**). The PCoA visualization revealed a separation of the data in three main groups: oropharyngeal region, nose and gut (ANOSIM, *p* < 0.05).

### Comparison with the HMP Dataset

**FIGURE 2:**
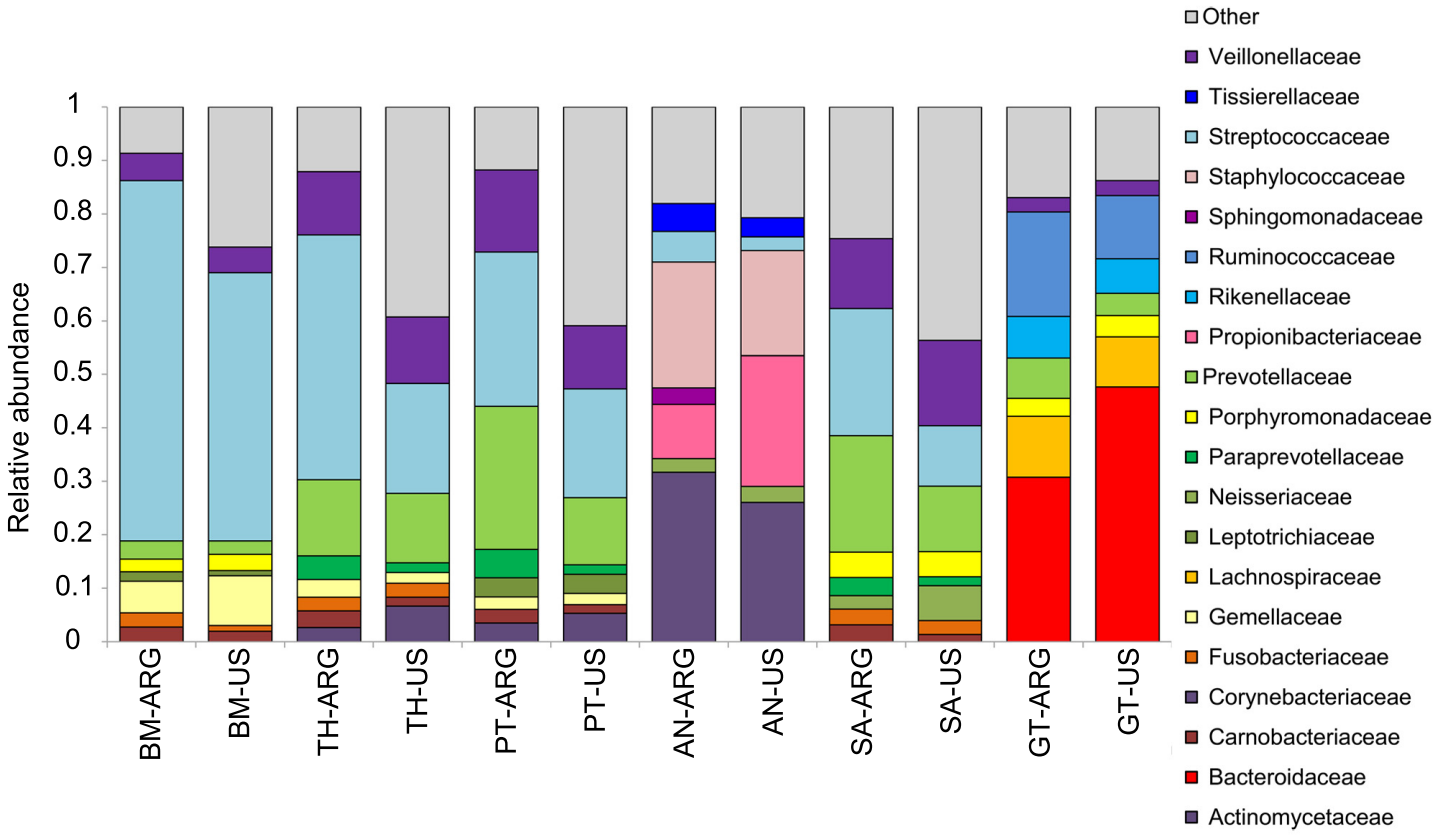
**Taxonomic profiles.** Twenty top abundant bacterial families in the Argentine and US (HMP) human microbiota. ARG, Argentine population; BM, buccal mucosa; TH, throat; PT, palatine tonsils; AN, anterior nares; SA, saliva; GT, gut.

We present here a simple analysis showing differences with the HMP dataset in order to encourage the use of the Argentine dataset for comparison purposes. We observed that the microbiota of the Argentine and US populations differed in composition and community structure. Beta diversity results based on weighted Unifrac distances showed that the microbiota of the buccal mucosa, the palatine tonsils, and the gastrointestinal tract differed between Argentine and US individuals (**Supplementary Figure S1**, ANOSIM, *p* < 0.05). Moreover, the taxonomic composition of these body habitats showed differences between populations. We observed differences in the abundance of the most predominant taxa (**Figure 2**). For example, the abundance of Bacteroidaceae family was higher in the US gut microbiota, while Ruminococcaceae, Lachnospiraceae, Rikenellaceae, and Prevotellaceae were more abundant in the Argentine gut microbiota. It is known that the variation in the levels of the three main taxa in the gut microbiota can define enterotypes (Arumugam et al., 2011). The higher relative abundance of three genera defines the enterotypes: bacteroides defines *enterotype 1*, Prevotella defines *enterotype 2* and of Ruminococcus defines *enterotype 3*. Although we found differences in Bacteroidaceae and Ruminococcaceae abundances (**Figure 2**, Mann–Withney *p* < 0.05) and Bacteroides, Ruminococcus, and Prevotella abundances (Mann–Withney *p* < 0.05, data not shown) between populations, both populations can be assigned to *enterotype 1* (**Supplementary Figure S2**). This is, to our knowledge the first time gut microbiota of middle-income populations of North and South America are compared. Differences in Prevotella and Bacteroides abundances between southamerican and US populations were previously reported. However, in these studies the microbiota of Ameridian individuals living in small villages near the Amazonas with the microbiota of western citizens living in metropolitan areas were compared (Yatsunenko et al., 2012). Regarding the buccal mucosa microbiota, we observed that Streptococcaceae family was more abundant in the Argentine population while Gemellaceae family was higher in the US individuals (**Figure 2**, Mann–Withney *p* < 0.05). The abundance of Streptococcaceae family was also higher in the palatine tonsils of argentine individuals. Our results encourage the idea that the human microbiota ecosystem has multiple states of equilibrium and that these variations are present between healthy populations. Moreover, it is probable that these multiple states are related to different lifestyles, location, ethnics, cultural tradition, age, and gender.

## Conclusion

Here, we present the first dataset based on human microbiota samples of an urban middle-income population in South America. We characterized the microbiota of six different body habitats: palatine tonsils, saliva, buccal mucosa, throat, anterior nares and gut from samples of healthy individuals living in a metropolitan area in Argentina. Our initial findings revealed differences in the structure and composition of the microbial communities compared to the US urban population.

By sharing our data, we want to actively encourage its reuse for comparison purposes. This will ultimately result in novel biological insights on the variability of the microbiota of healthy individuals across populations worldwide. Moreover, the understanding of the human microbiota ecosystem in a health-associated state will help to answer questions related to the role of the microbiota in disease.

### Data Access

Raw datasets are available in the NCBI SRA database under the accession number: SRP062999. Datasets can be downloaded as fasta or fastq files. The datasets are part of the NCBI Bioproject PRJNA293521^3^.

## Author Contributions

BC and MF analyzed the data and performed the numerical and statistical analyses. BC wrote the manuscript. SR processed the sequence raw data. MF and MS designed the protocol and criteria for healthy individual selection. MS wrote the protocol for recruiting the individuals, supervised the recruiting process, and collected the metadata for the individuals. GM performed DNA preparation from human samples. MR and BB performed amplicon library preparation and sequencing. FF directed the project and supervised the study. MV directed the project, conceived the study, supervised the bioinformatics analysis and supervised the manuscript.

## Conflict of Interest Statement

The authors declare that the research was conducted in the absence of any commercial or financial relationships that could be construed as a potential conflict of interest.

## Footnotes

2 ftp://public-ftp.hmpdacc.org/HMQCP/seqs_v13.fna.gz

3 http://www.ncbi.nlm.nih.gov/bioproject/PRJNA293521/
