# Supplementary material for: Human Microbiota of the Argentine Population- A Pilot Study
Source: Front Microbiol. 2016 Feb 1;7:51. doi: 10.3389/fmicb.2016.00051 (PMC4733923; doi:10.3389/fmicb.2016.00051)
Supplement: Supplementary file 1 [file Presentation_1.PDF]

Supplementary Material

Human Microbiota of the Argentine Population- A pilot study

Belén Carbonetto, Mónica Fabbro, Mariela Sciara, Analía Seravalle, Guadalupe Méjico, Santiago Revale, María Soledad Romero, Bianca Brun, Marcelo Fay, Fabián Fay, Martin Vazquez*.

*** Correspondence:** Corresponding Author: martin.vazquez@indear.com

Supplementary Figures and Tables

The following are the inclusion/exclusion criteria that subjects must have met to be recruited for this study:

Elegible criteria:

- Male or female subjects between 18 years and 50 years of age.
- Must be able to provide signed and dated informed consent.
- Healthy subjects willing and able to provide blood, as well as oral cavity, nasal cavity and stool specimens.

Exclusion Criteria:

Any subject who meets any of the following criteria will be excluded from participation in this study:

- Use of any of the following drugs within the last **6 months**:
  - systemic antibiotics, antifungals, antivirals or antiparasitics (intravenous, intramuscular, or oral);
  - oral, intravenous, intramuscular, nasal or inhaled corticosteroids;
  - cytokines;
  - methotrexate or immunosuppressive cytotoxic agents;
  - large doses of commercial probiotics consumed - includes tablets, capsules, lozenges, chewing gum or powders in which probiotic is a primary component. Ordinary dietary components such as fermented beverages/milks, yogurts, foods do not apply.
- Use of topical antibiotics or topical steroids on the face, scalp, or neck or on arms, forearms, or hands within the previous **7 days**.
- Acute disease at the time of enrollment (defer sampling until subject recovers). Acute disease is defined as the presence of a moderate or severe illness with or without fever.
- Chronic, clinically significant (unresolved, requiring on-going medical management or medication) pulmonary, cardiovascular, gastrointestinal, hepatic or renal functional abnormality, as determined by medical history or physical examination.

- Unstable dietary history as defined by major changes in diet during the previous month, where the subject has eliminated or significantly increased a major food group in the diet.
- Positive test for HIV, HBV or HCV.
- Any confirmed or suspected condition/state of immunosuppression or immunodeficiency (primary or acquired) including HIV infection.
- Major surgery of the GI tract, with the exception of cholecystectomy and appendectomy, in the past five years. Any major bowel resection at any time.
- History of active uncontrolled gastrointestinal disorders or diseases including:
    - inflammatory bowel disease (IBD) including ulcerative colitis (mild-moderate-severe), Crohn's disease (mild-moderate-severe), or indeterminate colitis;
    - irritable bowel syndrome (IBS) (moderate-severe);
    - persistent, infectious gastroenteritis, colitis or gastritis, persistent or chronic diarrhea of unknown etiology, *Clostridium difficile* infection (recurrent) or *Helicobacter pylori* infection (untreated);
    - chronic constipation.
- Regular urinary incontinence necessitating use of incontinence protection garments.
- Female who is pregnant or lactating.
- Chronic dry mouth, as assessed through questioning of the subject by an experienced clinician.
- Periodontal pockets equal to or greater than 4 mm. (Mild gingivitis is acceptable.)
- More than 10% of sites with bleeding on probing.
- Evidence of untreated cavitated carious lesions or oral abscesses.
- Evidence of precancerous or cancerous oral lesions.
- Evidence of oral candidiasis.
- Evidence of halitosis, as determined by organoleptic assessment by an experienced clinician.
- More than 8 missing teeth. The missing teeth must be due to 3rd molar extractions and/or teeth extracted for orthodontic purposes, teeth extracted as a result of trauma, or teeth that are congenitally missing.

Supplementary Table 1. Healthy individual's metadata. Data was collected by medical examination/interview. Individuals also donated blood to examine the presence of viral markers.

ID	2637818	2647790	2647811	2648948	2656020	2656738	2658057	2660455	2662754	2666201	2669870	2676265	2677314	2679211	2680411	2680493	2680783	2680782	2726173	2733675
**Gender**	F	F	M	M	F	M	M	F	F	F	M	F	M	F	M	M	M	F	F	M
**Age**	42	45	30	26	25	39	35	25	49	24	43	33	33	20	44	33	31	31	25	37
**Serologic Markers-Virus**																				
a-HIV	NEG	NEG	NEG	NEG	NEG	NEG	NEG	NEG	NEG	NEG	NEG	NEG	NEG	NEG	NEG	NEG	NEG	NEG	NEG	NEG
HBS Ag	NEG	NEG	NEG	NEG	NEG	NEG	NEG	NEG	NEG	NEG	NEG	NEG	NEG	NEG	NEG	NEG	NEG	NEG	NEG	NEG
HCV	NEG	NEG	NEG	NEG	NEG	NEG	NEG	NEG	NEG	NEG	NEG	NEG	NEG	NEG	NEG	NEG	NEG	NEG	NEG	NEG
VDRL	NEG	NEG	NEG	NEG	NEG	NEG	NEG	NEG	NEG	NEG	NEG	NEG	NEG	NEG	NEG	NEG	NEG	NEG	NEG	NEG
HCG	NEG	NEG			NEG			NEG	NEG	NEG		NEG		NEG				NEG	NEG	
**Buccal Screening**																				
Dry mouth syndrome	NO	NO	NO	NO	NO	NO	NO	NO	NO	NO	NO	NO	NO	NO	NO	NO	NO	NO	NO	NO
Dental abscesses	NO	NO	NO	NO	NO	NO	NO	NO	NO	NO	NO	NO	NO	NO	NO	NO	NO	NO	NO	NO
Cancerous or precancerous lesions	NO	NO	NO	NO	NO	NO	NO	NO	NO	NO	NO	NO	NO	NO	NO	NO	NO	NO	NO	NO
Candidiasis	NO	NO	NO	NO	NO	NO	NO	NO	NO	NO	NO	NO	NO	NO	NO	NO	NO	NO	NO	NO
Halitosis	NO	NO	NO	NO	NO	NO	NO	NO	NO	NO	NO	NO	NO	NO	NO	NO	NO	NO	NO	NO
Bleeding gums	NO	NO	NO	NO	NO	NO	NO	NO	NO	NO	NO	NO	NO	NO	NO	NO	NO	NO	NO	NO
# teeth	> 24	> 24	> 24	> 24	> 24	> 24	> 24	> 24	> 24	> 24	> 24	> 24	> 24	> 24	> 24	> 24	> 24	> 24	> 24	> 24
**Screening gastrointestinal**																				
Inflammatory bowel disease, ulcerative colitis, Crohn´s disease or indeterminate colitis	NO	NO	NO	NO	NO	NO	NO	NO	NO	NO	NO	NO	NO	NO	NO	NO	NO	NO	NO	NO
Irritable bowel syndrome	NO	NO	NO	NO	NO	NO	NO	NO	NO	NO	NO	NO	NO	NO	NO	NO	NO	NO	NO	NO
Bacterial gastroenteritis, colitis, gastritis, chronic diarrhea, recurrent infection by Clostridium difficile or Helicobacter Pylori	NO	NO	NO	NO	NO	NO	NO	NO	NO	NO	NO	NO	NO	NO	NO	NO	NO	NO	NO	NO
Chronic constipation	NO	NO	NO	NO	NO	NO	NO	NO	NO	NO	NO	NO	NO	NO	NO	NO	NO	NO	NO	NO

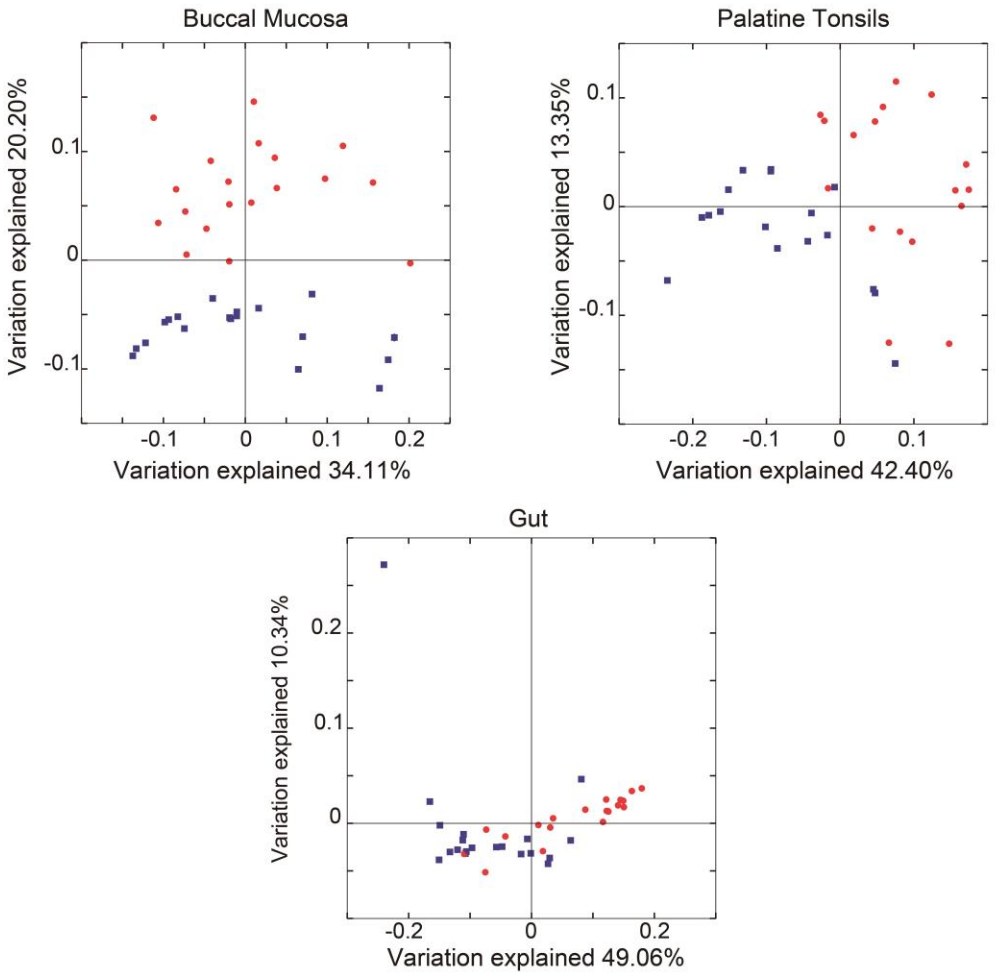

Supplementary Figure 1. Comparison of the Argentine vs. the US human microbiota. PCoA plots based on weighted Unifrac distances. Blue squares represent Argentine individuals and red dots represent US individuals.

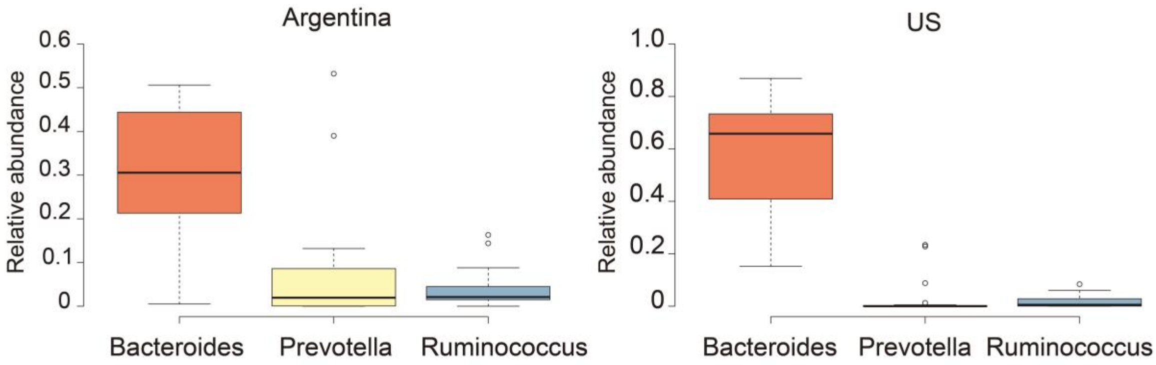

Supplementary Figure 2. Gut enteroypes. Taxa relative abundances.
